# Toxic tides of change: Ocean pollution as a cultural tipping point

**DOI:** 10.1007/s13280-025-02261-2

**Published:** 2025-10-16

**Authors:** Jessica M. Vandenberg, Eliana Ritts, Yoshitaka Ota, Russell Fielding

**Affiliations:** 1 Marine Affairs Department, University of Rhode Island, 202 Coastal Institute, 1 Greenhouse Rd, Kingston, RI 02881 USA; 2 HTC Honors College, Coastal Carolina University, PO Box 261954, Conway, SC 29528 USA; 3 Nippon Foundation Ocean Nexus, Kingston, USA

**Keywords:** Cultural keystone species, Environmental justice, Food sovereignty, Seafood, Slow violence, Sustainability

## Abstract

This paper offers a new perspective on the valuation of the impacts of industrial ocean pollution. Rising levels of industrial pollutants have a profound impact on marine resource-dependent peoples, particularly those dependent on seafood consumption. We argue that current regulation of these pollutants is both insufficient and inequitable, as it only accounts for impacts on physical health while ignoring cultural implications. This paper introduces the “cultural tipping point” as a new framework that integrates the impacts of ocean pollution on peoples’ physical and cultural health and well-being. Drawing on anthropology, marine sciences, public health, and critical Indigenous studies, the cultural tipping point synthesizes diverse concepts of “cultural keystone species,” food sovereignty, and industrial pollution. Ultimately, our goal is to make the cultural impacts of ocean pollution legible within global governance networks, and to advocate for greater allocation of societal resources to address this issue.

## Introduction

For peoples living in coastal and island settings, many marine species are more than a source of nutrition; they are part of people’s cultural worlds. These species, such as salmon for many Indigenous nations of the Pacific Northwest of Canada and the USA, or whales for Inuit communities across the Arctic, are deeply embedded in ceremony, cosmology, oral history, and kinship networks. Recognized as “cultural keystone species” (CKS), these plants and animals shape collective cultural identities and practices (Garibaldi and Turner 2004; Settee and Shukla 2020). Consumption of CKS grounds many areas of social reproduction, from kinship relations and ceremonial practices to political autonomy and economic sustainability (Coté 2022; Hutchings et al. 2022). For example, the return of salmon each year has traditionally marked the renewal of ecological and social cycles for Coast Salish peoples, bringing together communities in seasonal harvesting, feasting, and ceremony. In these settings, the annual return of the salmon is not just a matter of food, but is also a critical cultural practice that connects place, family, and community (Coté 2019). Through seasonal cycles, multispecies relationships, and ceremonial traditions, CKS are integral to how marine resource-dependent peoples worldwide remember their past, experience their present, and imagine their futures.

In this paper, we focus specifically on consumable CKS (i.e., species that are eaten) because they lie at the intersection of cultural and physical health, and are often the primary pathway through which toxic pollutants enter human bodies. This focus also reflects the urgent need to address a growing environmental crisis—the contamination of marine food sources by industrial pollutants. Escalating and emerging environmental changes, such as eutrophication and rising ocean temperatures, are putting marine-based food practices at unprecedented risk. Here, we draw particular attention to the rising threat of ocean pollution and seafood toxicity as a key area of anthropogenic change.^1^ Each year, an estimated 220 billion tons of industrial chemicals and heavy metals are released into the environment, primarily stemming from land-based activities such as mining, farming, construction, and energy production (Naidu 2021). Interdisciplinary studies demonstrate that seafood in some regions has reached pollution levels so high and acute that consumption poses physical health threats, including cognitive, reproductive, and developmental disorders, as well as premature deaths (Grandjean and Landrigan 2014; Underwood 2017; Leiser et al. 2019). As ocean pollution is anticipated to rise with increased industrial production, these health risks are only set to escalate (Naidu 2021). Moreover, climate change will amplify the effects of ocean pollution on marine species by increasing rates of ecological mobilization and biomagnification (Dudley et al. 2015). Such rapid growth in the production of polluting substances, coupled with insufficient control over their release, has expanded the impacts on human health from acute, localized issues to global, transboundary challenges.

In response to these challenges, recent studies have begun to consider the broader human impacts of ocean pollution, from food security (Golden et al. 2016) to disruption of traditional diets (Cisneros-Montemayor et al. 2016) and food systems (Kenny et al. 2021). However, the *cultural* impacts of ocean pollution—that is, impacts on the dynamic politics, knowledges, relations, values, materialities, and ways of making meaning that both shapes are shaped by peoples’ lives—have received less attention, particularly in the contexts of equitable ocean governance and environmental justice (Crossman et al. 2022). In this paper, we argue that it is crucial to account for these cultural impacts in order to more accurately and equitably assess the consequences of industrial ocean pollution. As long as cultural dimensions remain overlooked, acceptable levels of pollutant releases will continue to be inequitably overestimated by society. We propose the concept of a “cultural tipping point” as a tool to make these cultural impacts more visible and legible within global governance networks. While the term "tipping point" has its roots in describing significant, often abrupt transformations in ecological systems, we adapt the concept here to explore the profound cultural transformations that arise when marine resource-dependent communities are forced to navigate the complex trade-offs posed by toxic contamination of culturally significant “keystone” species. Through the cultural tipping point lens, we aim to draw attention to overlooked dimensions of ocean pollution and highlight systemic injustices in ocean governance that call for new forms of recognition and response.

## Defining a cultural tipping point

### Introducing tipping points: markers of social and ecological change

At the turn of the twenty-first century, climate and ecological scientists adopted the term “tipping point” to describe the rapid reconfiguration of ecosystems, moments when small changes produce abrupt and sometimes irreversible transformation, or “runaway change,” in habitat structure, species composition, and community dynamics (Lenton 2013; Serrao-Neumann et al. 2016; Van Nes et al. 2016).^2^ Such tipping points mark the threshold at which a system shifts from one state to another. Often referred to as a regime shift, the resulting reorganization of environmental systems is often difficult or even impossible to reverse due to reinforcing feedback loops (Folke et al. 2016). These shifts are rarely triggered by a single factor. Instead, they emerge from the compounding effects of multiple stressors, which may act gradually over time until an abrupt transition is triggered. Such drivers include climatic fluctuations, overexploitation, pollution, eutrophication, and invasive species (Andersen et al. 2008). For instance, the transformation of kelp forests to sea urchin barrens is linked to the climate change-driven range extension of sea urchins and the decline of predator species, disturbances that are only expected to increase in frequency (Marzloff et al. 2016). Not only do these ecological disturbances reflect biophysical processes, but they are also embedded in broader histories of capitalism and colonialism, resulting from human activities such as resource extraction practices, globalized trade networks, and industrialization (Hughes et al. 2013; Jouffray et al. 2020). Recognizing how these biophysical changes interact with social, political, and economic processes is critical for understanding root drivers of change and addressing degraded conditions (Nyström et al. 2012).

The previous studies have begun to connect environmental tipping points with social processes, specifically within the context of social–ecological systems (SES). SES frameworks posit that abrupt environmental changes affect the provision of ecosystem services, producing cascading effects on other social domains of the system (Lauerburg et al. 2020). Specifically, a “social tipping point” is defined as a point within an SES at which a small but measurable environmental change, maintained by a positive feedback mechanism, triggers a nonlinear change in the social component of the SES that inevitably and often irreversibly leads to a qualitatively different state of the social system (Milkoreit et al. 2018). Instances of social tipping points are largely examined through a historical lens, where a past ecological tipping point leads to significant changes in the cultural attributes of a society, or even major societal collapse (Fernández-Giménez et al. 2017). In contrast, research into present-day risks of SES reaching tipping points remains underdeveloped because studies on social tipping points tend to overlook socio-cultural factors within an SES (Lauerburg et al. 2020). While these studies focus on the livelihood impacts of ecological tipping points, cultural costs remain largely underexplored (Olsson et al. 2015).

### The “tipping point” reimagined: a cultural and political approach

Today, tipping points have captured the public imagination, popularized by Malcolm Gladwill and more recently taken up in the context of climate breakdown (Nuttall 2012). News headlines take up the theme of tipping points in increasingly apocalyptic terms (Skrimshire 2008), while 2022 saw the first Global Tipping Points Conference, resulting in a COP28 report and a follow-up conference scheduled for 2025 (Lenton et al. 2023). These popular discourses center urgency of action and narratives of no return. We join an emergent body of critique around these discourses, from Kyle White’s work (2019) on ecological and relational tipping points, showing how the guise of “urgency” is used to reinforce colonial harms against Indigenous peoples, to Mark Nuttall’s caution (2012) that adapting the “tipping point” from science to social sciences risks oversimplifying the complexity of human life to positivistic and predetermined models (2012). Max Liboiron questions the threshold thinking underlying tipping points, critiquing assimilationist theories that transform water into a resource for waste disposal (Liboiron 2021).

We share these concerns and, in this spirit, build on and extend existing conceptualizations of tipping points. By drawing attention to the cultural values at risk and the power dynamics at play, we use the cultural tipping point to argue for a fundamental shift in how pollution governance is approached: The need to be led by people most impacted, foregrounding their agency, experiences, and temporalities. While social–ecological systems frameworks have made important strides in integrating ecological and social dynamics, they often remain silent on the cultural ruptures and power dynamics that accompany environmental decline. We introduce the concept of a “cultural tipping point” to foreground these dimensions, drawing attention to the material, symbolic, and relational changes that occur when ecological harm undermines culturally significant species, practices, and identities. By highlighting interconnected impacts of pollution on diverse areas of human life, the cultural tipping point complicates and challenges contemporary governance regimes.

Cultural tipping points highlight intersecting areas of change in people's lives, connecting well-being with environmental shifts, public health, and relations with food sources. We define a cultural tipping point as the point at which continuing to consume a contaminated species would be detrimental to *physical* health and well-being, but ceasing consumption would be detrimental to *cultural* health and well-being. With this, we also recognize the economic and political processes that enable such tipping points to occur, including the powerful actors, policies, and environmental management systems that disproportionately place environmental injustices on those most vulnerable to ocean pollution. This definition brings together concepts from several distinct bodies of research: cultural keystone species, food sovereignty, Indigenous critical theory, and environmental justice. Each of these fields emphasizes different dimensions of human–nature relationships (i.e., the cultural values of species, the politics of food systems, the legacies of colonialism, and the uneven burden of environmental harms). Bringing these strands of literature together allows us to approach the cultural tipping point not only as an ecological, social, or health threshold, but also as a moment of cultural and political rupture and transformation. While our area of focus is ocean pollution, we suggest that this model has implications for other kinds of anthropogenic change, such as biodiversity loss and climate change.

To begin unpacking this framework, we first turn to the concept of “cultural keystone species,” or CKS. Although the past studies of social tipping points focus largely on broad shifts in natural resource-based livelihood strategies (Fernández-Giménez et al. 2017), *cultural tipping points* center CKS as culturally important food sources. Alternatively referred to as “culturally significant species” (IISAAK OLAM Foundation 2022), CKS are animals and plants that play a central role in the cultural identity, beliefs, and practices of a group of people (Garibaldi and Turner 2004). This concept is an adaptation of “ecological keystone species,” which describes an organism that plays a critical and singular role in an ecosystem, often disproportionate to its abundance (Coe and Gaoue 2020). CKS may double as ecological keystone species, but this is not always the case. CKS highlight a species’ roles across different aspects of human life, from diet, material resources, and economy to language, narratives, and ceremony (Garibaldi and Turner 2004; Indigenous Council of Experts 2018). Importantly, while some CKS are valued for their role in subsistence and food systems, others hold cultural significance primarily through non-consumptive relationships, such as spiritual symbolism, ceremonial use, or kinship with place and community. While there are many different ways of identifying CKS, the most direct way is through engaged, on-the-ground research, asking people which species they feel are key to their cultural and physical well-being, identity, and survival (Garibaldi and Turner 2004).^3^ Part of the power of the CKS framework is that it breaks down boundaries between “natural” and “human” worlds, emphasizing instead the interconnected, reciprocal relations between people, plants, animals, and place (Cuerrier et al. 2015). Examples include evergreen Fukugi trees in Japan (Chen and Akamine 2021), condors in the high Andes (Jacques-Coper et al. 2019), gray whales in the Pacific Northwest (Coté 2015), white sturgeon for Stó:lō peoples in Canada (Oloriz and Parlee 2020), and weed-eating pigs in central China (Wu 2021).

While not all CKS are consumable, many serve as food sources, and it is these species that we foreground in our discussion of cultural tipping points. To fully appreciate the significance of CKS as food, it is not enough to rely on dominant research that focuses on nutrition and food security. Such studies tend to neglect the complex networks of cultural relations that center food, undervaluing the multifaceted role of CKS in peoples’ lives (MacRae 2016; Alonso-Fradejas et al. 2017). We turn instead to an abundant body of work on food sovereignty and Indigenous food sovereignty, largely absent from SES writings on social–ecological tipping points, which offers more expansive understandings of CKS in relation to culture, practice, and place. Food sovereignty movements emerged as a reaction to industrialization, the power of corporate actors over land and resources, and transnational governance networks—all factors present in the ocean pollution problem. Most fundamentally, food sovereignty asserts peoples’ right to “healthy and culturally appropriate” foods, and the right to define their own food systems.^4^ Sovereignty offers a powerful political framework, moving beyond narrow framings of rights and access to directly address historical inequities and injustices. For instance, the Black food justice movement in the USA makes visible the racism and histories of slavery that shape anti-Black food systems, uplifting Black agriculture (White 2018) and Black food geographies (Reese 2019) as forms of resistance and co-liberation. As an international political movement (Alonso-Fradejas et al. 2017), food sovereignty is generative in the ways it transcends the nation-state, opening up radical possibilities for mobilization in global governance networks (Canfield 2022). As a result, food sovereignty highlights a potential justice-based governance framework that considers the multidimensional human–nature relations threatened by ocean pollution.

Food sovereignty is particularly important for Indigenous peoples around the world whose food systems have been disproportionately affected by ongoing processes of colonial dispossession and extraction (Daschuk 2019; Settee and Shukla 2020)—from the violences of residential schools (Talahongva 2018) in the USA to agricultural colonization in Aotearoa New Zealand (Hutchings 2025). Many Indigenous scholars and activists have adapted the concept to consider “Indigenous food sovereignty” specifically, centering Indigenous ecological knowledge, spirituality, self-determination, and ancestral wisdom (Salmón 2012; Coté 2022) as part of a broader project to decolonize diets (Mihesuah 2003). Since many of the coastal and ocean peoples who are impacted by ocean pollution identify as Indigenous, we foreground scholarship on Indigenous food sovereignty here to respect and incorporate a multiplicity of Indigenous perspectives. At the same time, while Indigenous food sovereignty is grounded in specific research contexts, this work has broader theoretical implications for understandings of food sovereignty more generally.

Theories of Indigenous food sovereignty take a holistic approach that embeds food in all aspects of life, from language and spirituality to political organization, intergenerational knowledge transmission, and ecological stewardship (Morrison 2011; Hutchings et al. 2022). People and CKS sustain each other through reciprocal relations of responsibility, kinship, and care (Kimmerer 2013). To highlight the practices and places at the heart of these relationships, some scholars alternatively describe CKS as “Cultural Keystone Species and Places” (CKSP) and highlight “Cultural Keystone Practices” like fire-burning undergrowth that sustain long-term connections between humans and CKS (IISAAK OLAM Foundation 2022). Practices of hunting, gathering, preparing, distributing, and eating CKS all serve to connect people with their human and non-human kin (Dawson 2020). For instance, for Seediq and Truku Indigenous peoples in Taiwan, hunting—particularly boar, muntjac, and sambal deer—is part of an extended moral and legal system through which people maintain their ethical responsibilities to humans in their community, humans in other communities, ancestors, and forest animals (Simon 2023). Such Indigenous-led approaches to food sovereignty reflect a profound perspectival shift from socio-ecological approaches to CKS to a more culturally-centered perspective that holds food, food-based practices, and places as more than resources. Rather, food is a relationship that protects and sustains the cultural agency of Indigenous and non-Indigenous peoples amid ongoing political and economic marginalization.

As food sovereignty studies decenter nutrition and security, they also offer an expanded concept of health and well-being (Arquette et al. 2002; Donatuto et al. 2011). Rather than treating “health” and “culture” as isolated categories, Indigenous approaches to food sovereignty integrate physical, emotional, psychological, cultural, and spiritual aspects of health and well-being (Van Oostdam et al. 2005). In the words of Nuu-chah-nulth First Nations scholar Charlotte Coté:

Being “healthy” is more than just our physical well-being; it means being spiritually and emotionally healthy—feeding our bodies with traditional and healthy foods and feeding our minds and spirits with our cultural teachings. For Indigenous people, our physical, spiritual, and emotional health is directly related to our ability to eat our traditional foods (2022, 37–38).

Coté rejects a narrow definition of “physical” health in favor of a more expansive, integrated understanding of well-being, centered around CKS. For Inuit peoples in northern Alaska, whale hunting, processing, and consumption provide subsistence and also build community solidarity, develop self-identity and self-esteem, strengthen ancestral connections, advance self-determination, and sustain cultural heritage (Freeman et al. 1998; Coté 2015). Meat and blubber from whales and other traditional foods are so inseparable from individual and collective well-being that a common saying goes: “I am what I am because of what I eat” (Coté 2015, 2022). These cultural and spiritual relations with food are profoundly political. Agency and access to consume CKS is shaped by larger power systems that govern important dimensions of human–nature relationships, such as who has access to which species and ecosystems, or which communities or ecologies are exposed to environmental harm—and who is responsible for causing harm.

What, then, happens at a cultural tipping point, when a cultural keystone species and food source becomes too toxic to safely consume? Loss of CKS has been linked with widespread loss of cultural practices, including food-focused collective labor and celebration, intergenerational transfer of knowledge, situational usage of traditional ecological knowledge, and opportunities for the use of specialized language (Van Oostdam et al. 2005). Examples include the impacts of the salmon fishery collapse on Indigenous groups in the Pacific Northwest of the USA (Colombi 2012), regulatory bans on the Inuit seal hunt in Canada (Dauvergne and Neville 2011; Rodgers and Scobie 2015), and regulatory bans on Makah whaling in the Pacific Northwest of the USA (Coté 2015). In the case of ocean pollution, contamination of cultural keystone seafood species has similarly widespread impacts across the communities that center and rely on them, as we demonstrate below. If only one dimension of these disruptions is considered—for instance, public health but not culture—then the overall impact of ocean pollution will be fundamentally undervalued. Moreover, while separating health and culture can be a useful methodological tool, it is out of sync with how people experience the impacts of contaminated CKS in their daily lives.

## Ocean pollution as a cultural tipping point driver

Over time ocean pollution can lead to cultural tipping points, at which continued consumption of culturally significant marine species would be detrimental to physical health, but ceasing to consume such CKS would have devastating cultural impacts. In other words, the point at which both the decision to and not to consume a contaminated species has negative consequences on physical and cultural health and well-being. Such tensions are heightened by the biophysical and chemical complexities that shape ocean pollution pathways. Ocean pollution is a diffuse and transboundary issue, where toxic substances originating from one place can have profound effects in other distant oceans. In recent years, pollutants such as nutrients, heavy metals, plastics, and synthetic chemicals have significantly increased in oceans and other aquatic ecosystems (Landrigan et al. 2020). As pollutants accumulate and biomagnify in marine food webs, consumption of marine species becomes a key pathway of human exposure. Pollutant concentrations among species and individual organisms may vary and are influenced by such factors as geographic origin, fish age, size, and species (Ueno et al. 2004; Nicklisch et al. 2017a, 2017b). Some marine mammals such as whales and dolphins are long-lived, sit high in marine food webs, and have large lipid deposits, which makes them particularly prone to accumulating high concentrations of certain pollutants (Schaap et al. 2023). This is especially concerning as many of these species are crucial CKS for various coastal and island communities globally, leaving them disproportionately vulnerable to the human health burdens associated with these pollutants. For example, elevated concentrations of persistent organic pollutants (POPs) have been detected in marine mammal-dependent populations such as Inuit men in Greenland (Lindh et al. 2012) and whaling communities in the Faroe Islands (Weihe et al. 2008).

Consumption of contaminated fish, shellfish, and marine mammals is the primary pathway humans are exposed to ocean pollution, leading to various health effects (Landrigan et al. 2020). For example, studies have shown that infant exposure to methylmercury, resulting from maternal consumption of contaminated seafood, is linked to significant neurological impairments and increased risks of autism, ADHD, and learning disorders (Debes et al. 2015; Julvez et al. 2019). Research with Canadian Inuit communities, who are exposed to relatively high levels of POPs through their traditional diet of fish, wild game, and marine mammals, found that this exposure is potentially linked to impairment of thyroid hormone systems and increased risk of type 2 diabetes (Château-Degat et al. 2010; Marushka et al. 2021). A study of Canadian Inuit children also revealed neuromotor impairments associated with postnatal exposure to seafood contaminants such as POPs and heavy metals (Boucher et al. 2016). Additionally, harmful algal blooms (HABs), which are triggered by pollutants such as phosphorus and nitrogen, produce toxins that accumulate in fish and shellfish, leading to severe neurological impairment when consumed (Berdalet et al. 2015). While it is clear that seafood consumption is a major pathway for exposure to toxic ocean pollutants, the full extent of its disease burdens remains largely unknown, with the exception of mercury (The Lancet Planetary Health 2019; Landrigan et al. 2020).

Beyond human health effects, ocean pollution can have profound consequences for coastal and marine resource-dependent communities, including losses in food sovereignty, livelihood, economics, culture, and biodiversity. Although research is limited, several previous studies have compellingly shown how these losses manifest across diverse cultural contexts and geographies. For instance, Coast Salish members of the Swinomish reservation in Washington State’s Puget Sound have faces difficult decisions around consumption of culturally significant shellfish (i.e., local species of clams, crabs, oysters, shrimp, and mussels) due to evidence of chemical contamination (Donatuto et al. 2011). With increasing levels of contaminants in shellfish, Swinomish people perceive threats to food sovereignty, ceremonial customs, traditional knowledge, and community cohesion (Donatuto et al. 2011). Similarly, Inuit communities in Canada face trade-offs related to consuming traditional “country” foods versus available store-bought foods (Kenny et al. 2021). Elevated levels of mercury and POPs in CKS such as ringed seals, narwhals, and beluga whales have prompted health advisories to reduce their consumption; however, diet transitions among Inuit peoples to energy-dense, micro-nutrient-poor store-bought foods have resulted in increased rates of cardiovascular disease and obesity (Sheikh et al. 2011). In New York state, the Mohawk Nation at Akwesasne is immediately downriver from two Superfund sites and adjacent to several other industrial projects, which have polluted the St. Lawrence River with PCBs since the 1950s. Over time, community members’ consumption of toxic fish and other aquatic animals has contributed to a wide range of health issues, including autoimmune dysfunctions (Lee et al. 2022). Resulting state and tribal advisories that warned against consumption of PCB-contaminated fish were controversial, perceived by some as eroding cultural identity (Schell et al. 2005).

As these examples start to make clear, pollution-driven dietary shifts are not just nutritional changes, but carry deep cultural consequences. Transitions away from CKS can disrupt culturally important food-based relations, including the loss of intergenerational knowledge and practices. At the same time, these cases also reflect how these patterns of lost CKS food systems are entangled with systems of colonial oppression and racial capitalism that are evident across all struggles over food sovereignty. Additional research has found elevated levels of contaminants across many other marine resource-dependent communities where traditional seafood diets are culturally significant among both Indigenous and non-Indigenous communities, including in Japan (Fujii et al. 2021), Taiwan (Chien et al. 2006), and the Faroe Islands (Mogensen et al. 2015). But while these studies continue to unfold the health effects related to consumption of contaminated seafood, the limited research on cultural impacts represents a significant gap in our understanding of how ocean pollution may drive cultural tipping points across diverse geographic and socio-cultural contexts.

Finally, even as pollution emerges as a critical driver of cultural tipping points for many coastal and marine resource-dependent communities, they remain difficult to trace. Unlike acute contamination events like oil spills, land-based sources of ocean pollution can represent a form of slow (and managed) violence “that occurs gradually and out of sight, a violence of delayed destruction that is dispersed across time and space, an attritional violence that is typically not viewed as violence at all” (Nixon 2011). This is partly due to the diffuse nature of these pollutants. Oceanic and atmospheric currents can carry pollutants globally, enabling long-range transport to remote parts of the world, including the Arctic and Antarctic regions (AMAP 2018). Temporally, slow violence necessitates examining risks or threats beyond those that are immediate and obvious to health and safety. Contamination of cultural keystone marine species may unfold slowly over time. As a result, impacts on marine ecosystems tend to be identified retrospectively rather than in real-time (Marzloff et al. 2016).

We find slow violence a generative concept in the ways it invites us to examine experiences of violence not as isolated present-day incidents, but instead as legacies of historical processes of extraction and inequity (Nixon 2011; Liboiron 2021; Fuller 2022). In other words, slow violence expands how we understand “harm” beyond the temporal and material limits that often bound this concept. By recognizing ocean pollution as a form of slow violence, we can start to discern the actors and actions responsible for harm that were previously obscured, whether by the extensive lag time between cause and effect, or by what Auyero and Swistun (2009) describe as the “labor of confusion”: the production and management of uncertainties that mediate and manage public understandings of ocean pollution. Governance actors such as corporations and governments may offer conflicting information about contamination or, in more extreme cases, collude to deliberately obscure its consequences. Such labor of confusion can amplify uncertainties around toxicity thresholds and risks, rendering ocean pollution an “invisible threat” despite firsthand accounts from affected communities of its gradual effects on daily life (Johansen 2023). Davies (2018) draws attention to the political implications of this work, arguing that toxic slow violence persists because it occurs in populations whose stories do not “count,” and therefore are vulnerable to sacrifice. This underscores the significance of recognizing ocean pollution as a critical yet often overlooked driver of cultural tipping points, requiring governance and regulatory actions that prioritize justice.

As toxic uncertainty and the slow, diffuse violence that characterizes ocean pollution make it difficult to track and advocate for transformative governance actions, vulnerable communities are left to make difficult decisions around the trade-offs of consuming contaminated CKS. Many people in coastal and island communities experience cultural tipping points as a kind of high-stakes moral choice, a trade-off between two negative outcomes: should they stop consuming a contaminated CKS to the detriment of their social, cultural, psychological, and spiritual well-being, or should they continue consumption to the detriment of their physical health? Faced with chemical contamination of culturally significant shellfish in Puget Sound, a Swinomish elder explained: “It’s our spiritual food so it feeds our soul; so it might poison our body, but then we’d rather nourish our soul” (Donatuto et al. 2011). To return to Akwesasne, even though consumption advisories have resulted in overall decreases in levels of PCBs, these changes are not viewed as a success story. Research conducted by the Akwesasne Task Force on the Environment alongside scientists at the University of Albany shows how these regulations fail to account for the ways that not consuming fish jeopardizes cultural survival and integrity (Schell et al. 2005). In their words: “being asked to avoid activities that reaffirm Mohawk identity is not a solution to this problem but [is] a bigger problem in and of itself” (Schell et al. 2005).

These examples show how the “choice” to consume or not to consume contaminated CKS is not really a choice at all, but creates a false sense of agency that shifts responsibility away from the industries and governing bodies responsible for ocean pollution in the first place. Who produces the toxins that contaminate marine ecosystems? Who regulates the pollutants that make marine CKS so dangerous to consume? The answers are rooted in histories of colonial and capitalist extraction, which disproportionately impact coastal and island peoples (many of whom are Indigenous) who are dependent on marine resources. Laying bare these politics makes clear: Ocean pollution as a driver of cultural tipping points is an issue of environmental justice and must be taken seriously as such on a global level. To reach any transformative pathway of effective governance or to avoid the ongoing “slow violence” of ocean pollution, we must recognize the need to deconstruct our values and prejudice that have rendered ocean pollution burdens invisible at every level of governance.

## Conclusion

In this paper, we highlight the need to consider culture when examining the consequences of ocean pollution, particularly for coastal and marine resource-dependent communities. The risks of ocean pollution are currently undervalued because the consequences of ocean pollution remain narrowly defined. While physical health impacts are increasingly documented, broader effects on other aspects of life and well-being, including culture, are insufficiently considered in current environmental risk assessments and ocean pollution management policies. We demonstrate that this results in insufficient regulation of pollutants at local and global levels, shifting the burden of managing pollution from responsible state and non-state actors to impacted peoples. Through the cultural tipping point, we have laid out a framework that emphasizes the intricate and often overlooked connections between ocean pollution and coastal and island peoples through marine-based food systems. For many of these communities, declining access to seafood due to ocean pollution not only represents the loss of a local food source but also a profound loss of identity and culture. These losses must be accounted for when evaluating the true costs of ocean pollution.

Ultimately, our objective is to intervene in global ocean governance networks by making visible some of the overlooked aspects of industrial ocean pollution. We integrate interdisciplinary perspectives to offer a new model of cultural and ecological change, demonstrating that the impacts of ocean pollution on health and culture are inseparable. As a strategic framework, the cultural tipping point pressures those involved in ocean pollution governance to address long-standing, systemic injustices by holding non-state corporate actors and state actors accountable for their role in the production of pollutants through cooperative international policy.

Drawing attention to the cultural impacts of ocean pollution strengthens the argument for increased regulation of industrial pollutants. Furthermore, case studies of cultural tipping points can offer supporting evidence to advocate for greater allocation of societal resources toward identifying the presence of ocean pollutants and potential harms associated with their release, as well as toward protecting marine cultural keystone species. Supporting networks of place-based, local movements of ocean pollution justice is critical to ensuring that the voices of those most impacted are raised and the lived realities of ocean pollution consequences are recognized in all of their complexity.

This work comes with complex challenges—many of which stem from the temporally and spatially diffuse nature of the pollutants in question, complicating efforts to track, regulate, and assign responsibility. The transboundary nature of ocean pollution allows polluters and pollutants to evade national jurisdictions and oversight, undermining enforcement and disavowing accountability. In many cases, it is unclear who has the authority, capacity, or political will to respond, particularly when pollutants cross multiple regulatory jurisdictions or venture into international waters. These challenges mirror other transboundary ocean governance issues, including what are increasingly referred to as “blue crimes,” such as illegal and unregulated fishing or waste dumping. Like ocean pollution, these activities exploit legal loopholes, weak enforcement, and fragmented ocean governance regimes. Addressing the cultural tipping points triggered by ocean pollution, therefore, requires us to understand not only the pollutants themselves but also the systemic governance failures that allow these harms to accumulate unchallenged.

Another layer of interdisciplinary collaboration is needed to understand how to intervene, partnering with public policy and legal scholars to map the policy landscape, identify key actors, and develop strategies to shift the power imbalance currently existing between polluters and those suffering from the consequences. This is also where food sovereignty, as we discussed above, comes back into play, not only as a way to understand the cultural impacts of ocean pollution but also as an alternative model of resistance that transcends the nation-state and better aligns with current global governance networks (Canfield 2022). As such, food sovereignty provides a potential path for an equitable and just governance framework that considers the multitude of human–nature relations and values threatened by ocean pollution.

Finally, we hope that the cultural tipping point will highlight what is at stake in global conversations around industrial ocean pollution: human dignity, food sovereignty, and environmental justice. As many of the marine resource-dependent peoples who are most impacted by these pollutants have experienced centuries of colonial, imperial, and capitalist extraction, the measurement and regulation of ocean pollution become issues of environmental justice and social inequity. These histories of marginalization are amplified by contemporary research and governance practices, which continue to neglect peoples’ cultural values in assessing the risks and impacts of industrial pollutants. The burden of managing the effects of industrial pollutants thus falls largely on impacted peoples, rather than those responsible for producing or regulating the emissions. We argue that as long as cultural impacts remain sidelined in global governance settings, the full extent of ocean pollution will remain undervalued, and the historic injustices rooted in colonial and capitalist systems of extraction will persist. The cultural tipping point offers a framework to imagine more just and equitable futures of human–animal–ocean networks at multiple levels of scale. For while we highlight here the significance of localized lifeways, ocean pollution has always been and will continue to be a global issue. In the words of Inuk activist Sheila Watt-Cloutier: “We are the miner’s canary. It is only a matter of time until everybody will be poisoned by the pollutants that we are creating in this world.”

## Footnotes

1 A note on terminology: Among the many forms of ocean pollution, we focus on pollutants that stem from industrial activities (i.e., fossil fuel chemical production and use, industrial agriculture, mining, etc.), because the accumulation of these pollutants poses the highest risks to seafood toxicity. Unless otherwise specified, whenever we use the term “ocean pollution,” we are referring to these kinds of industrial pollutants.

2 The concept of a tipping point originates from late nineteenth century mathematics and chemistry, where it described a type of qualitative, systemic change known as bifurcation (for a full history of “tipping points,” see Milkoreit et al. 2018).

3 Other criteria include (1) intensity, type, and multiplicity of use; (2) abundance; (3) naming and terminology; (4) irreplaceability; (5) use in trade or resource acquisition; (6) psycho-socio-cultural function (e.g., symbolism, knowledge transmission, etc.); (7) a high level of importance (Coe and Gaoue 2020).

4 These rights were established through the World Forum for Food Sovereignty in 2007 as the Nyéléni Declaration, a document that aimed to establish an international process to formally recognize the right to food sovereignty.
